# Transcriptional progressive patterns from mild to severe renal ischemia/reperfusion-induced kidney injury in mice

**DOI:** 10.3389/fgene.2022.874189

**Published:** 2022-07-22

**Authors:** Dong Lai, Lei Wang, Jia-Rui Li, Chen Chen, Wen-Lei Zhao, Qing Yuan, Xin Ma, Xu Zhang

**Affiliations:** ^1^ Department of Urology, The Third Medical Center, Chinese PLA General Hospital, Beijing, China; ^2^ Biomedical Innovation Center, Beijing Shijitan Hospital, Capital Medical University, Beijing, China

**Keywords:** renal ischemia/reperfusion injury, time series analysis, dynamic network biomarker analysis, progressive patterns of I/R kidney injury, gene set enrichment analysis

## Abstract

The renal ischemia/reperfusion (I/R)-induced acute kidney injury incidence after nephron-sparing surgery for localized renal tumors is 20%, but the biological determinant process of postoperative acute kidney injury remains unclear. Using Gene Expression Omnibus database (GSE192883) and several bioinformatics analyses (discrete time points analysis, gene set enrichment analysis, dynamic network biomarker analysis, etc), combined with the establishment of the I/R model for verification, we identified three progressive patterns involving five core pathways confirmed using gene set enrichment analysis and six key genes (*S100a10*, *Pcna*, *Abat*, *Kmo*, *Acadm*, and *Adhfe1*) verified using quantitative polymerase chain reaction The dynamic network biomarker (DNB) subnetwork composite index value is the highest in the 22-min ischemia group, suggesting the transcriptome expression level fluctuated sharply in this group, which means 22-min ischemia is an critical warning point. This study illustrates the core molecular progressive patterns from mild to severe I/R kidney injury, laying the foundation for precautionary biomarkers and molecular intervention targets for exploration. In addition, the safe renal artery blocking time of nephron-sparing surgery that we currently accept may not be safe anymore.

## Introduction

To date, nephron-sparing surgery (NSS) has been the standard treatment for localized renal tumors (Campbell et al., 2009), and offers equivalent oncologic outcomes (Scosyrev et al., 2014) with better preservation of renal function (Capitanio et al., 2015) compared with radical nephrectomy. Renal ischemia/reperfusion (I/R)-induced acute kidney injury (AKI) manifests as a rapid decline in renal function after NSS and is associated with high mortality and an increased risk of chronic kidney disease (Bedford et al., 2012; Birnie et al., 2014; Chawla et al., 2014).

Approximately 20% of patients develop AKI after NSS, which is mainly caused by an insufficient compensatory capacity of the contralateral kidney (Simmons et al., 2013; Bravi, 2020). However, the impact on renal function may be hidden by compensatory mechanisms in the contralateral kidney and evolve into unilateral kidney atrophy (Lane et al., 2011; Porpiglia et al., 2012). Therefore, it is important to study the biological progressive patterns from mild to severe I/R kidney injury to determine the occurrence of postoperative AKI.

Ischemic time has been associated with the degree of postoperative kidney injury in mice; a 22-min ischemia time leads to restorable kidney injury, and a 25/30-min ischemia time leads to non-restorable kidney injury (Wei and Dong, 2012; Liu et al., 2017). For humans, recommendations suggest that ischemia time should be controlled to 20–25 min (Thompson et al., 2010). Different ischemic times result in different degrees of kidney injury in different patients. Therefore, studying the molecular mechanism of renal I/R injury is the key to accurately evaluating the potential degree of kidney injury and to discovering potential molecular targets.

Previous studies have focused on the mechanisms of severe kidney injury and recovery after reperfusion, and current mainstream theories include cell death, inflammatory response, and fibrous repairing (Kumar et al., 2014; Venkatachalam et al., 2015; Cippa et al., 2018). For sequencing-related studies, Andrew et al. revealed molecular characterization of the transition from acute to chronic kidney injury following I/R by studying changes in molecular patterns, including cell death and proliferation (*Krt8*, *Krt20*, and *Sox9*), cell cycle and wound repair (*Havcr1* and *Lcn2*), cell adhesion and inflammation (*Timp2*), adaptive immune responses (*Ptprc*, *Cd3d*, *Cd74*, and *Cd48*) and tubular function (*Kap* and *Lrp2*) (Liu et al., 2017). They clearly revealed the molecular progressive patterns from AKI to chronic kidney disease after renal I/R but did not focus on molecular changes in mild I/R kidney injury or the progressive patterns from mild to severe I/R kidney injury.

I/R injury is a cascade amplification process that involves progressive patterns from mild to severe I/R kidney injury. Unlike previous studies investigating a single molecule or several molecules, it seems that progressive patterns from mild to severe I/R kidney injury are more conducive for urologists to understand postoperative AKI and explore precautionary markers, even monitoring the progress of molecular biology in real time during NSS.

Herein, we used RNA sequencing to detect transcriptome expression in continuous ischemic time groups from mild to severe injury in a mouse I/R model as reported previously (Battistone et al., 2020). The observed transcriptional differences were reproduced using specific gene and protein expression analyses. We found gene sets with significant changes in mild and severe kidney injury and described and verified the progressive pattern from mild to severe injury through functional enrichment analysis, discrete time points analysis (Kumar and Matthisa, 2007), dynamic network biomarker (DNB) (Liu et al., 2014), and gene set enrichment analysis (GSEA) (Subramanian et al., 2005), laying the foundation for further research.

## Materials and methods

### Animals

All animal experiments were performed in accordance with international guidelines and approved by the Institutional Animal Care and Use Committee of the PLA General Hospital. Male C57BL/6 mice aged 6–8 weeks were purchased from GemPharmatech Co., Ltd. Mice were housed in a pathogen-free, constant temperature environment with a 12 h light/dark cycle and allowed to acclimatize for a week in the animal facility before the operation.

### Ischemic renal injury model

Mice were subjected to bilateral renal I/R injury as previously described (Zhou et al., 2012). Briefly, mice were anesthetized with isoflurane on a thermostatic blanket while maintaining the temperature of the mice at approximately 37°C. Bilateral renal pedicles were dissociated through a middle abdominal incision, and the bilateral renal pedicles were clamped with atraumatic vascular clips for 18 or 30 min (n = 3). The incision was closed in two layers, and the mice were injected subcutaneously with 0.3 ml of warm saline on the back after surgery for volume supplementation. Sham-operated mice underwent the same procedure but without clamping of the renal pedicles.

### Bioinformatics analysis

The edgeR package from Bioconductor was used to explore DEGs. DEGs were analyzed using the edgeR package (Robinson et al., 2010). DEGs were finally determined using parameters of false discovery rate adjusted *p* value <0.01, and log2 fold change >1.2 or < −1.2 unless specified. The Mfuzz (Kumar and M) package from Bioconductor was used for the discrete time points analysis. GO and KEGG analyses were performed to enrich the biological processes and molecular pathways of different gene sets (Ashburner et al., 2000; Yamada et al., 2006). GSEA was used to identify the activation or inhibition of the screened pathways (Subramanian et al., 2005). Enrichment *p*-values were adjusted using the Benjamini and Hochberg method, and p-adjusted values of less than 0.05 were determined to be significantly enriched.

DNB analysis was performed as previously reported (Ideker and Sharan, 2008; Jin et al., 2008; Liu et al., 2014). DNB is a dynamic analysis conducted through the variation of genes themselves and the correlation between genes, A gene group with larger variation and less correlation with other genes means that the gene group has outlier performance and becomes unstable, thus determining that the biological process is transforming to another stage. When another stage of the disease is reached, this phenomenon disappears. Briefly, we selected genes with significantly high deviations at each time point. Next, we built a distance matrix with Pearson correlation coefficients (*PCC*) for each pair of genes and set a threshold of 0.9 to group genes. The composite index (CI) was calculated from the average standard deviation (SD) of DNB molecules, the average absolute PCC value inside the DNB cluster (PCC_1_), and the average absolute PCC value of the DNB cluster and others (PCC_0_), as follows:

$$CI=\frac{PCC_{i}}{PCC_{0}}SD$$

### Quantitative RT-PCR assays

After 24 h of reperfusion, the kidneys of all mice were used for RNA extraction. TRIzol Reagent (Invitrogen, Carlsbad, CA, United States) was used in accordance with the manufacturer’s instructions, and reverse transcription (RT) was performed using the QuantiTect Reverse Transcription Kit. The cDNA samples were used for gene expression quantification via real-time PCR with a 2x Super SYBR Green qPCR Master Mix purchased from ESscience (ES-QP002, Shanghai, China), and RT-PCR was performed using an Applied Biosystems QuantStudio3 (Applied Biosystems). Each gene was run in three technical replicates and normalized to Ppia, and the fold-change relative to the sham group was calculated using the 2^−ΔΔCt^ formula. Primers for RT-PCR were designed using Primer-BLAST (NCBI).

### Statistical analysis

Each analysis was performed in at least three independent experiments. All data are presented as the mean ± SEM (standard error of the mean). Differences between two groups were determined by two-tailed Student’s t-test using the Prism five software (GraphPad, La Jolla, CA, United States). Two-way analysis of variance (ANOVA) was used for analysis between the three groups. Statistical significance was set at *p* < 0.05.

## Results

### Differentially expressed genes and discrete time points analysis

**FIGURE 1:**
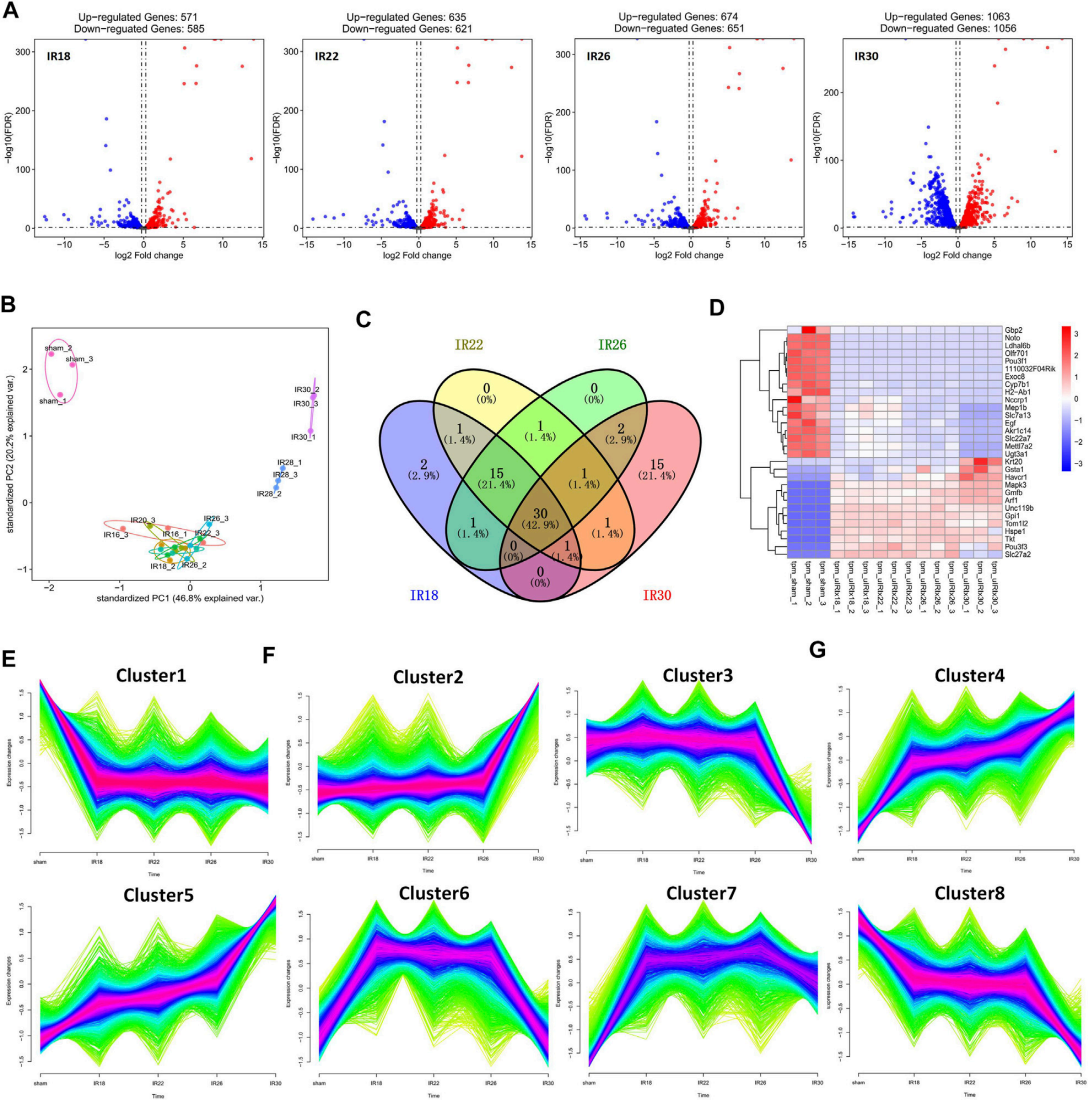
Description of differentially expressed genes (DEGs) of four ischemic time groups. **(A)** Volcano plot and **(B)** principal component analysis (PCA) for the DEGs of sham, IR18, IR22, IR26, IR30 group, which show that the number of DEGs increased with the extension of ischemia time, and that there is a significant difference between mild injury and severe kidney injury. **(C)** Venn diagram of top 50 DEGs from each group, reflecting that the difference is most obvious in IR30 **(D)** Heat map of overlapping DEGs among the four experimental groups, showing that the expression level of these DEGs changes regularly as the injury intensifies. **(E)** Cluster1 obtained by clustering indicates that the change of expression level was constantly accompanied by injury, which was a gene set related to ischemia/reperfusion (I/R) injury. **(F)** Cluster2 and Cluster3 show gene sets that change only in severe injury, and represent the gene sets related to severe I/R injury **(G)** Cluster4–8 represent gene sets with significant changes in expression level during the transition from mild to severe kidney injury, and which are related to the progressive patterns from mild to severe kidney injury.

To identify the differentially expressed genes at various times after renal I/R in mice, we performed RNA sequencing to explore the transcriptional progressive patterns from mild to severe kidney injury using different ischemia-time treatments. RNA sequencing data were deposited into the Gene Expression Omnibus database (GSE192883). Using differential gene analysis, we found that compared with the sham group, there were 1,156 differentially expressed genes (DEGs; 571 upregulated and 585 downregulated) in the 18-min ischemia group, 1,256 DEGs (635 upregulated and 621 downregulated) in the 22-min ischemia group, 1,325 DEGs (674 upregulated) in the 26-min ischemia group, and 651 DEGs (downregulated) and 2,119 DEGs (1,063 upregulated and 1,056 downregulated) in the 30-min ischemia group (Figure 1A). Among them, the top 10 DEGs with the greatest differences were more than 9-fold upregulated or downregulated (Table 1). Combined with principal component analysis (Figure 1B), it can be concluded that a large number of DEGs exist not only in severe renal I/R injury but also in mild renal I/R injury, indicating that many biological processes have been initiated in mild renal I/R injury and continue to change with the aggravation of the injury.

**TABLE 1:** Top 10 differentially expressed genes (DEGs) in four ischemic time groups.

Group	Gene symbol	logFC	PValue	FDR	Location (mouse)
**IR18**	Gmfb	13.88	0	0	Chromosome 14, NC_000080.7
	Pou3f3	13.59	2.21E-121	4.83E-119	Chromosome 1, NC_000067.7
	Olfr701^a^	-12.49	3.83E-22	1.91E-20	Chromosome 7, NC_000073.7
	Hspe1	12.44	1.36E-278	4.83E-276	Chromosome 1, NC_000067.7
	Noto	-12.29	9.61E-17	3.29E-15	Chromosome 6, NC_000072.7
	Ldhal6b	-10.07	1.73E-25	1.04E-23	Chromosome 17, NC_000083.7
	Mapk3	9.78	0	0	Chromosome 7, NC_000073.7
	Pou3f1	-9.50	3.62E-17	1.32E-15	Chromosome 4, NC_000070.7
	Unc119b	9.07	0	0	Chromosome 5, NC_000071.7
	Tkt	8.89	0	0	Chromosome 14, NC_000080.7
**IR22**	Noto	-14.15	5.47E-18	2.02E-16	Chromosome 6, NC_000072.7
	Gmfb	13.77	0	0	Chromosome 14, NC_000080.7
	Pou3f3	13.76	3.97E-125	8.05E-123	Chromosome 1, NC_000067.7
	Olfr701^a^	-12.50	3.67E-22	1.68E-20	Chromosome 7, NC_000073.7
	Hspe1	12.40	3.75E-276	1.33E-273	Chromosome 1, NC_000067.7
	Pou3f1	-11.33	1.08E-19	4.24E-18	Chromosome 4, NC_000070.7
	Ldhal6b	-10.08	1.63E-25	8.92E-24	Chromosome 17, NC_000083.7
	Mapk3	9.83	0	0	Chromosome 7, NC_000073.7
	Unc119b	8.99	0	0	Chromosome 5, NC_000071.7
	Tkt	8.88	0	0	Chromosome 14, NC_000080.7
**IR26**	Olfr701^a^	-14.36	1.63e^−23^	7.13e^−22^	Chromosome 7, NC_000073.7
	Noto	-14.15	5.53e^−18^	1.64e-^16^	Chromosome 6, NC_000072.7
	Gmfb	13.93	0	0	Chromosome 14, NC_000080.7
	Pou3f3	13.55	1.30e^−120^	2.84e^−118^	Chromosome 1, NC_000067.7
	Hspe1	12.45	4.37e^−279^	1.77e^−276^	Chromosome 1, NC_000067.7
	Ldhal6b	-11.39	6.39e^−28^	3.56e^−26^	Chromosome 17, NC_000083.7
	Pou3f1	-11.33	1.08e^−19^	4.09e^−18^	Chromosome 4, NC_000070.7
	Mapk3	9.73	0	0	Chromosome 7, NC_000073.7
	Tkt	9.02	0	0	Chromosome 14, NC_000080.7
	Unc119b	8.90	0	0	Chromosome 5, NC_000071.7
**IR30**	Olfr701^a^	−14.36	1.59e^−23^	1.20e^−22^	Chromosome 7, NC_000073.7
	Gbp10^a^	−14.31	5.64e^−18^	3.11e^−17^	Chromosome 5, NC_000071.7
	Gmfb	14.28	0	0	Chromosome 14, NC_000080.7
	Noto	−14.15	5.44e^−18^	3.00e^−17^	Chromosome 6, NC_000072.7
	Pou3f3	13.35	3.40e^−116^	7.44e^−114^	Chromosome 1, NC_000067.7
	Hspe1	12.28	1.19e^−269^	4.85e^−267^	Chromosome 1, NC_000067.7
	Ldhal6b	−10.26	7.10e^−26^	6.06e^−25^	Chromosome 17, NC_000083.7
	Mapk3	10.18	0	0	Chromosome 7, NC_000073.7
	Pou3f1	−9.90	8.26e^−18^	4.48e^−17^	Chromosome 4, NC_000070.7
	Tgtp1^a^	−9.15	7.01e^−23^	5.09e^−22^	Chromosome 11, NC_000077.7

a Represent genes specific to mice.

Among the top 50 DEGs in each group, the proportion of overlapping genes between the 18-min and 22-min ischemia groups was 67.1%; the proportion of overlapping genes among the 18-min, 22-min, and 26-min ischemia groups was 64.3%; and the proportion of overlapping genes among the four experimental groups was 42.9%. This suggests that DEGs gradually changed with the aggravation of injury but the most different genes changed unsustainably and discontinuously as time changes (Figure 1C). The heat map of overlapping DEGs among the four experimental groups shows that the expression levels of these DEGs changed regularly as the injury intensified (Figure 1D), supporting the results of the discrete time points analysis.

During the time-series analysis, all genes were automated and clustered into eight groups. Cluster1 indicated that the change in expression level was constantly accompanied by injury, which was a gene set related to I/R injury (Figure 1E). Cluster2 and Cluster3 showed gene sets that changed only in severe injury and represented the gene sets related to severe I/R injury (Figure 1F). Cluster4 to Cluster8 gene sets relate to the progression from mild to severe I/R kidney injury and showed a trend of continuous upregulation (Cluster4 and Cluster5), upregulation followed by downregulation (Cluster6 and Cluster7), and continuous downregulation (Cluster8). Moreover, the 18-min and 30-min ischemia groups were the two time points with more obvious changes, suggesting progressive patterns from mild to severe renal I/R injury (Figure 1G). Phenotypically, the injury initially occurred in the IR30 group but not the IR18, IR22 or IR26 group suggests that the transcriptome in the IR18 group has undergone changes, while they are not reflected in the pathological sections (Supplementary Figure S4). The results of the Cluster1–8 Kyoto Encyclopedia of Genes and Genomes (KEGG) and Gene Ontology (GO) functional enrichment analyses are presented in Table 2.

**TABLE 2:** Kyoto Encyclopedia of Genes and Genomes (KEGG) and Gene Ontology (GO) functional enrichment analysis of eight clusters obtained from discrete time points analysis.

Cluster	Description (KEGG)	GeneRatio	Pvalue	Description (GO)	Gene Ratio	pvalue
Cluster1	Antigen Processing and presentation	33/728	2.74e^−14^	Defense response to virus	61/1,660	3.04E-18
	Allograft rejection	27/728	7.15e^−14^	Response to symbiont	61/1,660	3.04E-18
	Graft-versus-host disease	26/728	6.13e^−13^	Response to interferon-beta	25/1,660	5.31E-17
Cluster2	Estrogen signaling pathway	30/854	6.84e^−06^	Leukocyte migration	74/2,175	9.44E-11
	MAPK signaling pathway	52/854	7.76e^−06^	Wound healing	73/2,175	9.55E-11
	Oxytocin signaling pathway	32/854	1.53e^−05^	Regulation of vasculature development	63/2,175	7.86E-10
Cluster3	Oxidative phosphorylation	54/713	3.21e^−25^	Oxidative phosphorylation	44/1,639	2.95E-23
	Chemical carcinogenesis-reactive oxygen species	63/713	5.77e^−20^	ATP metabolic process	66/1,639	6.76E-21
	Thermogenesis	59/713	2.19e^−16^	NADH dehydrogenase complex assembly	28/1,639	3.65E-20
Cluster4^a^	Ribosome	57/872	1.16e^−16^	mRNA processing	105/1924	8.13E-22
	Spliceosome	47/872	8.97e^−16^	Ribonucleoprotein complex biogenesis	91/1924	9.28E-19
	Nucleocytoplasmic transport	39/872	2.30e^−12^	ribonucleoprotein Complex subunit organization	53/1924	4.80E-17
Cluster5^a^	*Salmonella* infection	69/984	2.67e^−13^	Oxidative phosphorylation	44/1,639	2.95E-23
	*Yersinia* infection	44/984	7.74e^−12^	ATP metabolic process	66/1,639	6.76E-21
	Fc gamma R-mediated phagocytosis	33/984	2.38e^−10^	NADH dehydrogenase complex assembly	28/1,639	3.65E-20
Cluster6^a^	Cell cycle	26/689	2.37e^−06^	Chromosome segregation	85/1711	3.01E-25
	Oxidative phosphorylation	27/689	3.37e^−06^	Nuclear chromosome segregation	70/1711	1.75E-21
	Thermogenesis	34/689	0.000178	Nuclear division	91/1711	3.12E-20
Cluster7^a^	DNA replication	20/621	6.18e^−15^	DNA repair	109/1,445	1.35E-32
	Mismatch repair	12/621	3.86e^−09^	DNA replication	62/1,445	2.98E-21
	Cell cycle	28/621	2.13e^−08^	DNA-dependent DNA replication	46/1,445	3.67E-21
Cluster8^a^	Valine, leucine and isoleucine degradation	28/753	7.22e^−16^	Organic acid catabolic process	65/1,669	8.06E-25
	Propanoate metabolism	19/753	1.66e^−13^	Small molecule catabolic process	76/1,669	2.11E-21
	Peroxisome	31/753	7.13e^−13^	Fatty acid metabolic process	87/1,669	2.69E-20

a Represent pathways involved in progressive patterns from mild to severe kidney injury.

### GSEA and qPCR verification

**FIGURE 2:**
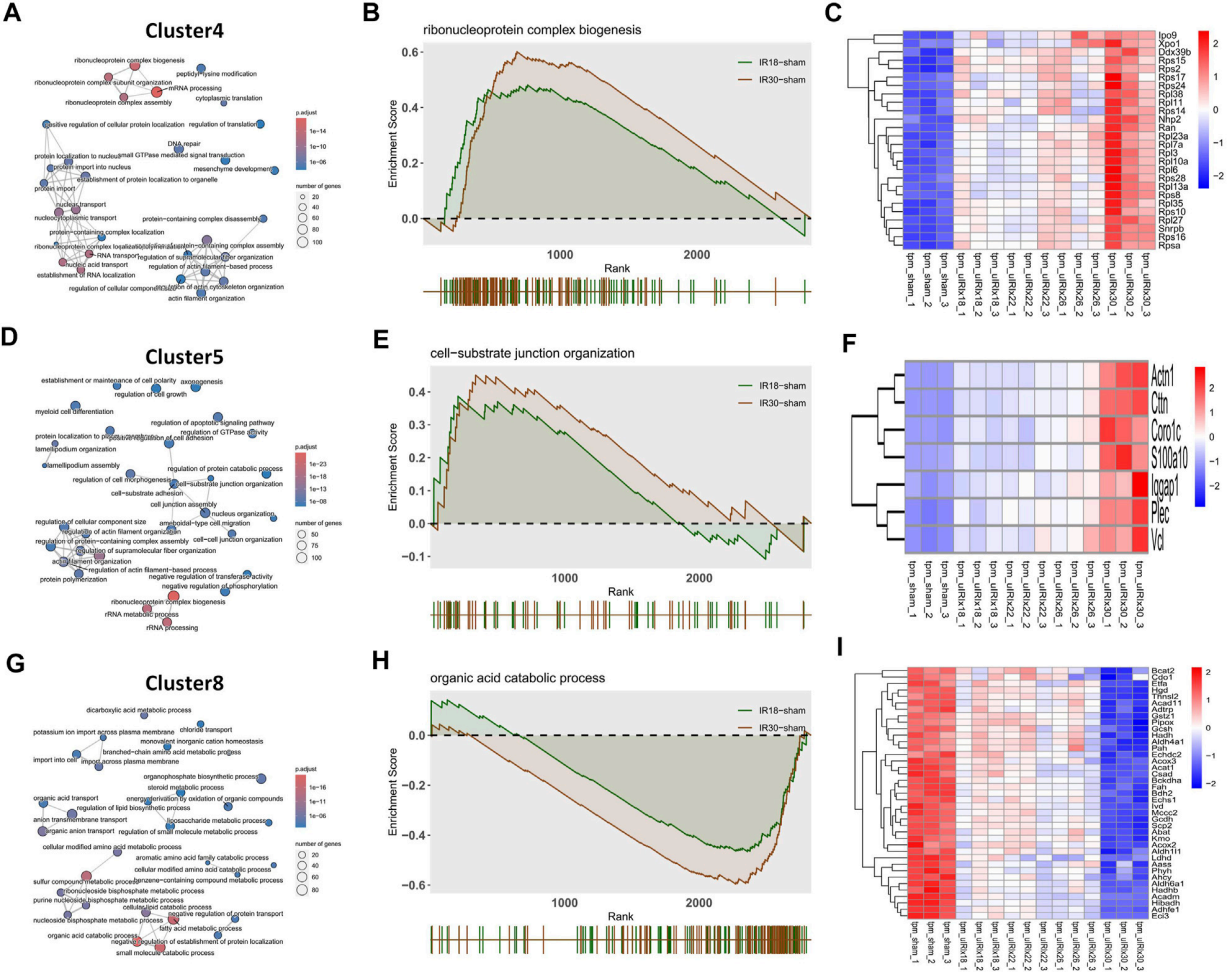
Discrete time points analysis and gene set enrichment analysis (GSEA) with gene heat maps. **(A)** Cluster4 **(D)** Cluster5, and **(G)** Cluster8 show “Ribonucleoprotein complex biogenesis”, “Cell-substrate junction organization”, and “Organic acid catabolic process” core pathways, respectively. **(B, E, and H)** GSEA analysis of cluster4, Cluster5, and Cluster8 core pathways, which may play a core role during the transition from mild to severe ischemia/reperfusion (I/R) kidney injury and **(C, F, and I)** heat maps of gene expression profiles enriched in these pathways, respectively.

The core pathway of Cluster4 was identified as “Ribonucleoprotein complex biogenesis” through the overlapping pathways and pathway network analysis (Figure 2A) of the 18-min and 30-min ischemia groups; the GSEA result (Figure 2B) showed that the activation level of this pathway in the 30-min ischemia group was higher than that in the 18-min ischemia group. This suggests that the biogenesis function of the ribonucleoprotein complex was activated during the transition from mild to severe I/R kidney injury. The heat map of core enrichment genes, which contributed the most to the enrichment results, showed the same transitional pattern (Figure 2C). The core pathways of Cluster5 and Cluster8 were identified as “Cell-substrate junction organization” and “Organic acid catabolic process” through the same method as Cluster4 (Figures 2D,G); GSEA results (Figures 2E,H) showed that the activation level of “Cell-substrate junction organization” in the 30-min ischemia group was higher than that in the 18-min ischemia group and the inhibition level of “Organic acid catabolic process” in the 30-min ischemia group was higher than that in the 18-min ischemia group. This indicates that the adhesive function between the cell and substrate was activated and that the organic acid catabolic process was inhibited during the transition from mild to severe I/R kidney injury.

**FIGURE 3:**
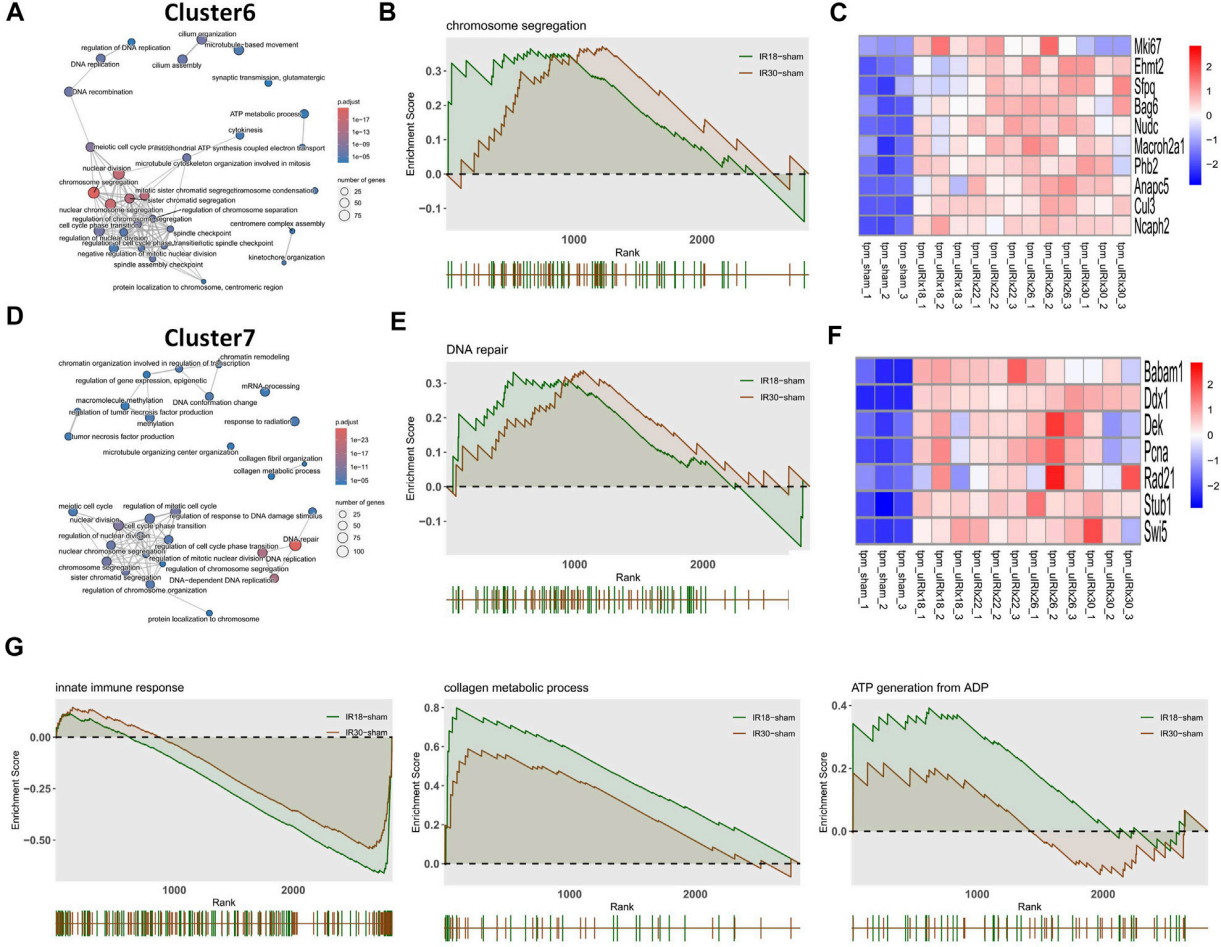
Discrete time points analysis and gene set enrichment analysis (GSEA) with gene heat maps. **(A)** Cluster6 and **(D)** Cluster7 show “Chromosome segregation” and “DNA repair” core pathways, respectively. **(B,E)** GSEA analysis of Cluster6 and Cluster7 core pathways, which may play a core role during the transition from mild to severe ischemia/reperfusion (I/R) kidney injury and **(C,F)** heat maps of gene expression profiles enriched in these pathways, respectively. **(G)** Representative pathways of GSEA enrichment pathways in other clusters.

**FIGURE 4:**
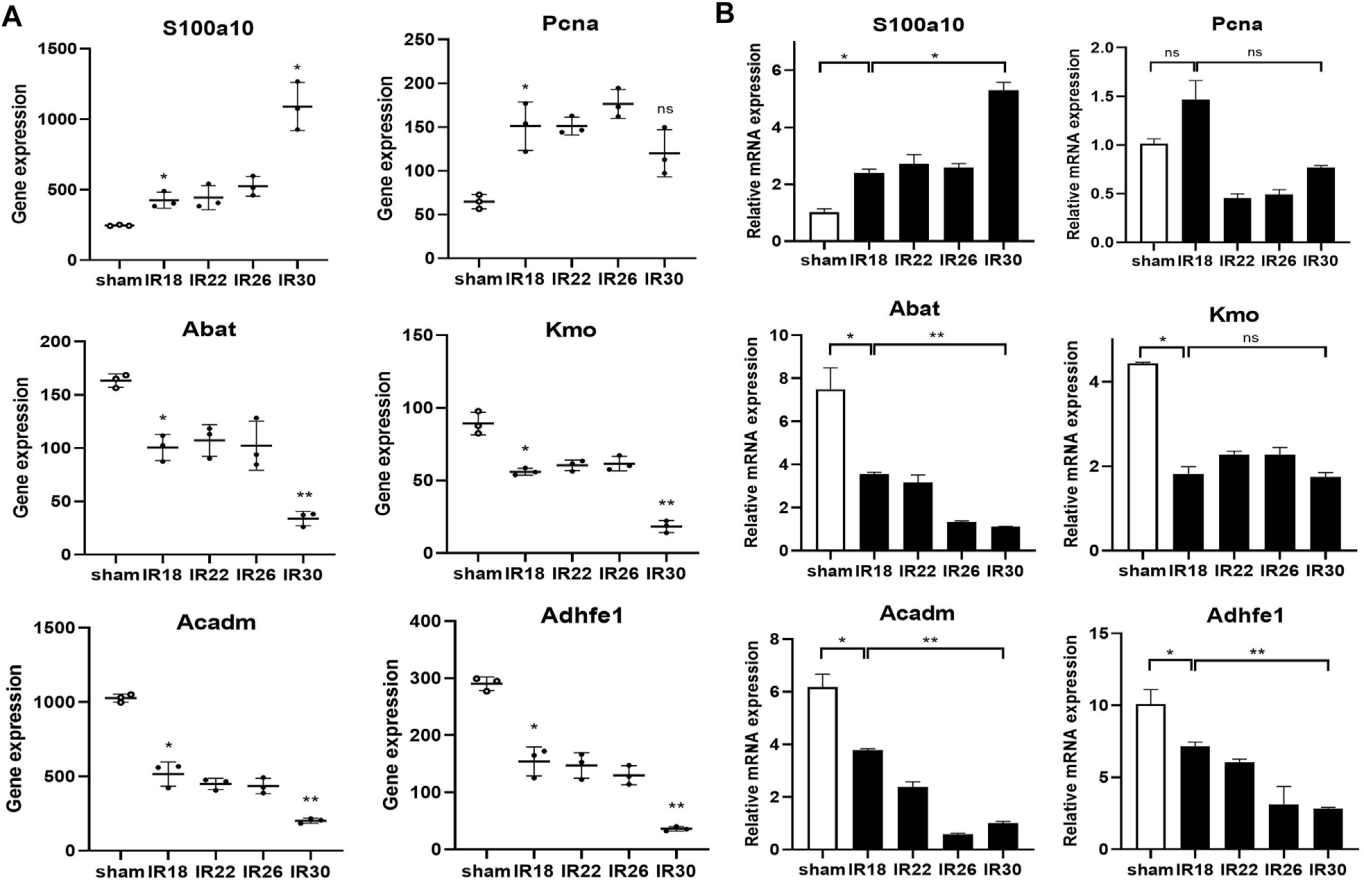
qPCR validation and sequencing results of key genes in Cluster4–8. **(A)** Sequencing results of key molecules of core pathways in five clusters, * represents P < 0.05, ** represents P < 0.01, the hollow circle represents the control group **(B)** Corresponding qPCR verification results showing the key molecules involved in the transition from mild to severe ischemia/reperfusion (I/R) kidney injury and potential targets for interventions.

The heat map of the core enrichment genes showed the same transitional pattern (Figures 2F,I). The core pathways of Cluster6 and Cluster7 were identified as “Chromosome segregation” and “DNA repair” through the same method (Figures 3A,D), and the GSEA results (Figures 3B,E) showed that there were no significant differences between the activation level of these two pathways in the 18-min and the 30-min ischemia groups; however, in the leading edge subset, the pathway activation level of the 18-min group was significantly higher than that of the 30-min group. The heat map shows a similar transitional pattern (Figures 3C,F). The other representative pathways of the five clusters with significant differences, as well as the representative pathways of other clusters, are also shown (Figure 3G, Supplementary Figures S1, S2). Genes with a limit fold-change (LFC) greater than 0.7 in the IR18 group, LFC greater than one in the IR30 group or LFC greater than one in the IR18 group, and LFC greater than 0.7 in the IR30 group were screened out in the leading edge subset of each core pathway as key genes (Supplementary Figure S3), and quantitative polymerase chain reaction (qPCR) verification was conducted. The results showed that *S100a10* was continuously upregulated in the IR18 and IR30 groups, which is consistent with the sequencing results. *Pcna* was upregulated in the IR18 group and downregulated in the IR30 group. *Abat, Kmo, Acadm*, and *Adhfe1* were continuously downregulated (Figure 4B), which is consistent with the sequencing results for these molecules (Figure 4A). However, there were no significant changes between IR18, IR22 and IR26 groups.

### DNB analysis

**FIGURE 5:**
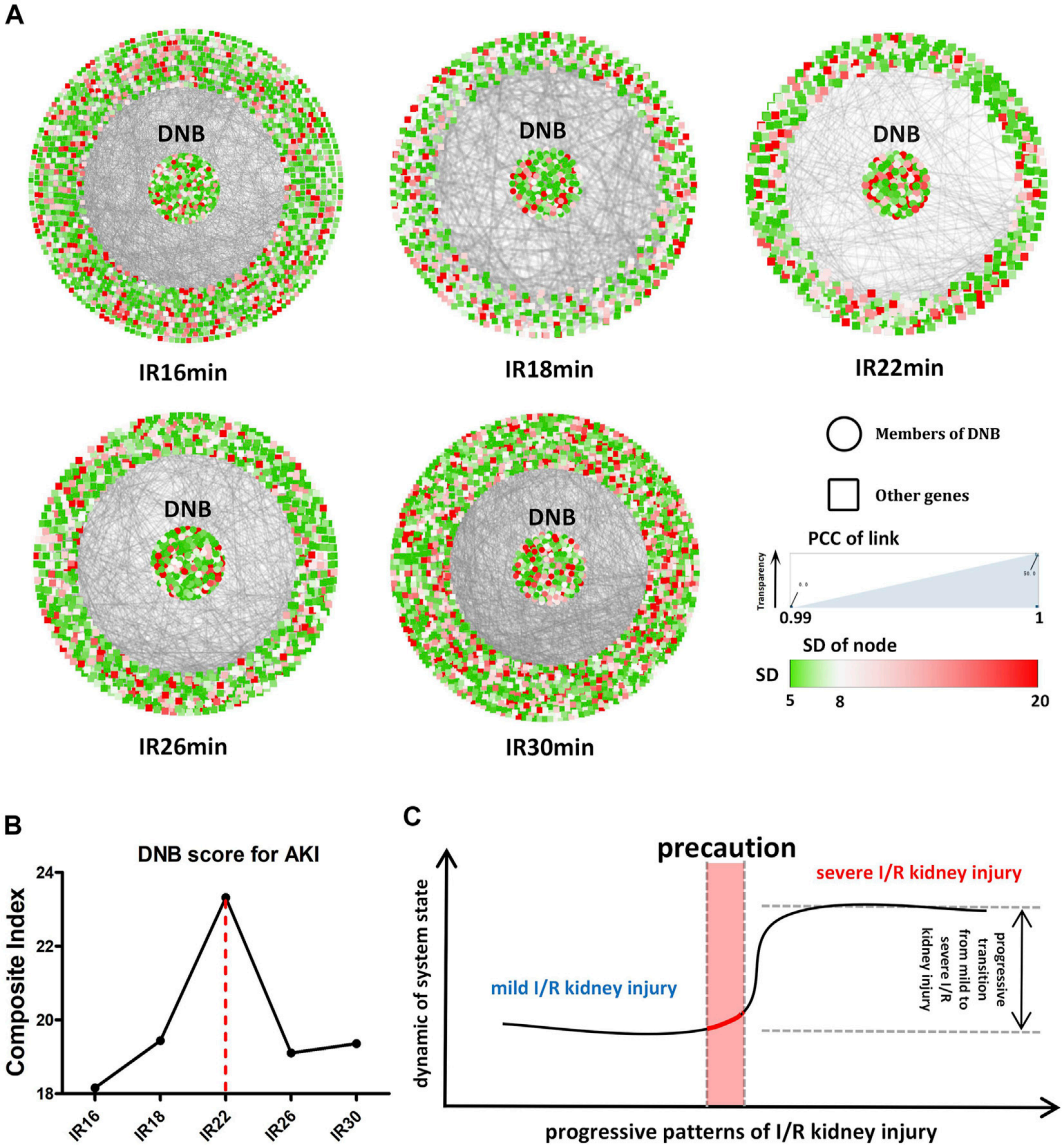
Dynamic network biomarker (DNB) analysis. **(A)** Situation of the whole molecular network with the prolongation of ischemia time in different ischemic groups. The DNB subnetwork is located in the center of the circle, and other genes are located around the periphery. The color of each dot represents the standard deviation (SD) value of the gene, and the transparency of the line between dots represents the correlation between the two genes. **(B)** CI (composite index) values of different ischemic groups, among which CI values of the 22-min ischemic group reach the peak. **(C)** Transition from mild to severe ischemia/reperfusion (I/R) kidney injury, suggesting the existence of a “pre-disease” state in renal I/R models.

Specifically, we used a time-course (16–30 min) of mice for the I/R injury with a renal pedicle clip. Using our sequencing data, we obtained gene subnetworks (DNB) with fluctuations in different ischemic time groups according to DNB analysis methods. The CI value of the DNB subnetwork was the highest in the 22-min ischemia group, suggesting that the transcriptome expression level fluctuated sharply in this group and that 22 min of ischemia may be the key time point in the transition from mild to severe I/R kidney injury. When the ischemia time was prolonged and the injury was aggravated, the CI value of DNB began to decline, confirming that DNB only specifically recognizes the state of “pre-disease”, as previously reported (Liu et al., 2014) (Figure 5B). The molecular network diagram shows that except for the 22-min ischemia group, DNB in the other ischemic groups had strong connections with other genes, indicating that the internal network resonance of DNB in this group was strong, while its relationship with other genes was significantly weakened (Figure 5A). Detailed calculation results are shown in Table 3. However, the standard deviation and internal correlation of DNB in the 22-min ischemia group were not the highest, suggesting that the key contribution to the transition from mild to severe injury during renal I/R lies in the weakened relationship between DNB and other genes. Briefly, during the transition from mild to severe I/R kidney injury, renal transcriptome expression levels fluctuated strongly when the ischemia time reached 22 min, and the injury began to transition from mild to severe (Figure 5C). Therefore, 22 min of ischemia can be considered a warning point for severe irreversible I/R kidney injury.

**TABLE 3:** Composite index (CI) calculation related indicators of the dynamic network biomarker (DNB).

Group	selected_gene_number	mean_SD	pcc1	pcc0	CI
IR16	2,232	10.53	0.97	0.56	18.15
IR18	832	10.63	0.97	0.53	19.43
IR22	745	10.69	0.96	0.44	23.32
IR26	1,288	10.24	0.96	0.51	19.10
IR30	2065	11.18	0.97	0.56	19.35

## Discussion

AKI can be caused by a variety of diseases, such as sepsis (Hoste et al., 2015), cardiorenal syndrome (McCullough et al., 2013), hepatorenal syndrome (Amin et al., 2019), and cardiac surgery-associated AKI(Hundemer et al., 2021). Different diseases have different characteristics in the process that leads to AKI. For urology, NSS-related I/R induced kidney injury is possessed of “controllability” and “monistic nature,” because the ischemia time in NSS is controllable, and the injury factor is relatively single compared with other diseases. Therefore, urologists should pay more attention to progressive patterns from mild to severe I/R kidney injury than to progressive patterns of kidney injury after I/R. This study focused on the progressive patterns from mild to severe I/R kidney injury after NSS, which provides a novel direction for monitoring and early warning of severe renal I/R injury, as well as the potential molecular targets for extending the time window of controllable kidney injury and striving for precious operation time for surgeons.

Previous studies on NSS-related AKI have suggested that ischemia time and renal tolerance to ischemia are decisive factors for the occurrence of postoperative AKI(Yossepowitch et al., 2006; Iida et al., 2008), but the internal molecular mechanism has not yet been explored. Most studies on molecular mechanisms have focused on biological changes in the kidney after reperfusion and the molecular or signaling pathways that play key roles in them. For example, *KIM-1* is significantly upregulated in injured proximal renal tubular epithelial cells, and plays an important role in the clearance of necrotic tubular epithelial cell debris; it thus affects the repair process of renal injury (Yang et al., 2015). Many studies have been conducted on innate immunity-related molecules, such as *IL-34, P2Y14*, and *PP2Acα,* which play important roles in the process of renal injury and repair by regulating the function of macrophages and neutrophils (Baek et al., 2015; Battistone et al., 2020; Liang et al., 2021).

In contrast to the above studies, we propose a progressive pattern in which the core pathway best reflects the transition from mild to severe I/R kidney injury. Although these pathways may not play a decisive role in I/R injury and repair, they and the molecules involved may play a crucial role in determining the degree of I/R kidney injury. In other words, the core pathway involved in the pattern shown in our study is a key criterion for evaluating the degree of I/R kidney injury, and may also be an important target molecule that slows down the process from mild to severe I/R kidney injury. *Pcna* and *Kmo* have been reported to be closely associated with renal I/R injury and may be used as standards to evaluate the degree of injury and as targets for molecular intervention in the future (Li et al., 2017; Zheng X. et al., 2019).

However, the remaining four molecules have not yet been reported, among which *S100a10* is associated with cell adhesion to the matrix and is also involved in the regulation of cell cycle and differentiation. *Abat*, *Acadm,* and *Adhfe1* are metabolism-related molecules, among which *Abat* and *Adhfe1* are involved in the metabolism of butyrate in cells. According to literature reports, butyrate metabolism is closely related to I/R kidney injury (Zheng Y. et al., 2019; Sun et al., 2022); therefore, *Abat* and *Adhfe1* may also play an important role in I/R kidney injury, suggesting that they can be used as important indices to evaluate the potential degree of I/R kidney injury and important molecular targets for intervention. Meanwhile, for therapy, the downregulation of *Abat* and *Adhfe1* leads to butyrate metabolic imbalances, which significantly enhanced renal dysfunction and histologic damage induced by renal IRI. Repairing metabolic imbalances through interventions can help increase butyrate which can cause a significant attenuation of neutrophil infiltration, which was reflected by the reduction of renal MPO activity, reduce apoptotic tubular cell death and improve caspase-3 activation. Therefore, Abat and Adhfe1 protein may be a potential therapeutic agent for preventing renal IRI (Zheng Y. et al., 2019).

Additionally, DNB analysis was introduced in this study to verify the existence of a “pre-disease” state through a new theory in renal I/R models, and it is proposed that before the occurrence of biological progressive patterns, the organism has already sent signals (DNB violent fluctuation). This provides a new idea for clinical warning, monitoring, and intervention for renal I/R injury. In addition, the safe renal artery blocking time of nephron-sparing surgery that we currently accept may not be safe anymore. However, our study also has some limitations. For example, the number of mice in each group was not sufficiently large, which may have caused bias in the analysis process. At the verification level, only RNA expression was verified, and further in-depth and comprehensive studies are required.

## Data Availability

The datasets presented in this study can be found in online repositories. The names of the repository/repositories and accession number(s) can be found in the article/Supplementary Material.
