# Corrigendum: Transcriptional progressive patterns from mild to severe renal ischemia/reperfusion-induced kidney injury in mice

**DOI:** 10.3389/fgene.2022.1011276

**Published:** 2022-09-13

**Authors:** Dong Lai, Lei Wang, Jia-Rui Li, Chen Chen, Wen-Lei Zhao, Qing Yuan, Xin Ma, Xu Zhang

**Affiliations:** ^1^ Department of Urology, The Third Medical Center, Chinese PLA General Hospital, Beijing, China; ^2^ Biomedical Innovation Center, Beijing Shijitan Hospital, Capital Medical University, Beijing, China

**Keywords:** renal ischemia/reperfusion injury, molecular progressive pattern, discrete time points analysis, gene set enrichment analysis, dynamic network biomarker analysis

In the published article, there was an error in the **author list**, and author Lei Wang was not labeled as the co-first author. The corrected author list appears below.

“Dong Lai1^†^, Lei Wang1^†^, Jia-Rui Li2, Chen Chen2, Wen-Lei Zhao1, Qing Yuan1*, Xin Ma1*, and Xu Zhang1*”.

The authors apologize for this error and state that this does not change the scientific conclusions of the article in any way. The original article has been updated.

