# Supplementary figures and images for: Transcriptional progressive patterns from mild to severe renal ischemia/reperfusion-induced kidney injury in mice
Source: Front Genet. 2022 Jul 22;13:874189. doi: 10.3389/fgene.2022.874189 (PMC9355309; doi:10.3389/fgene.2022.874189)

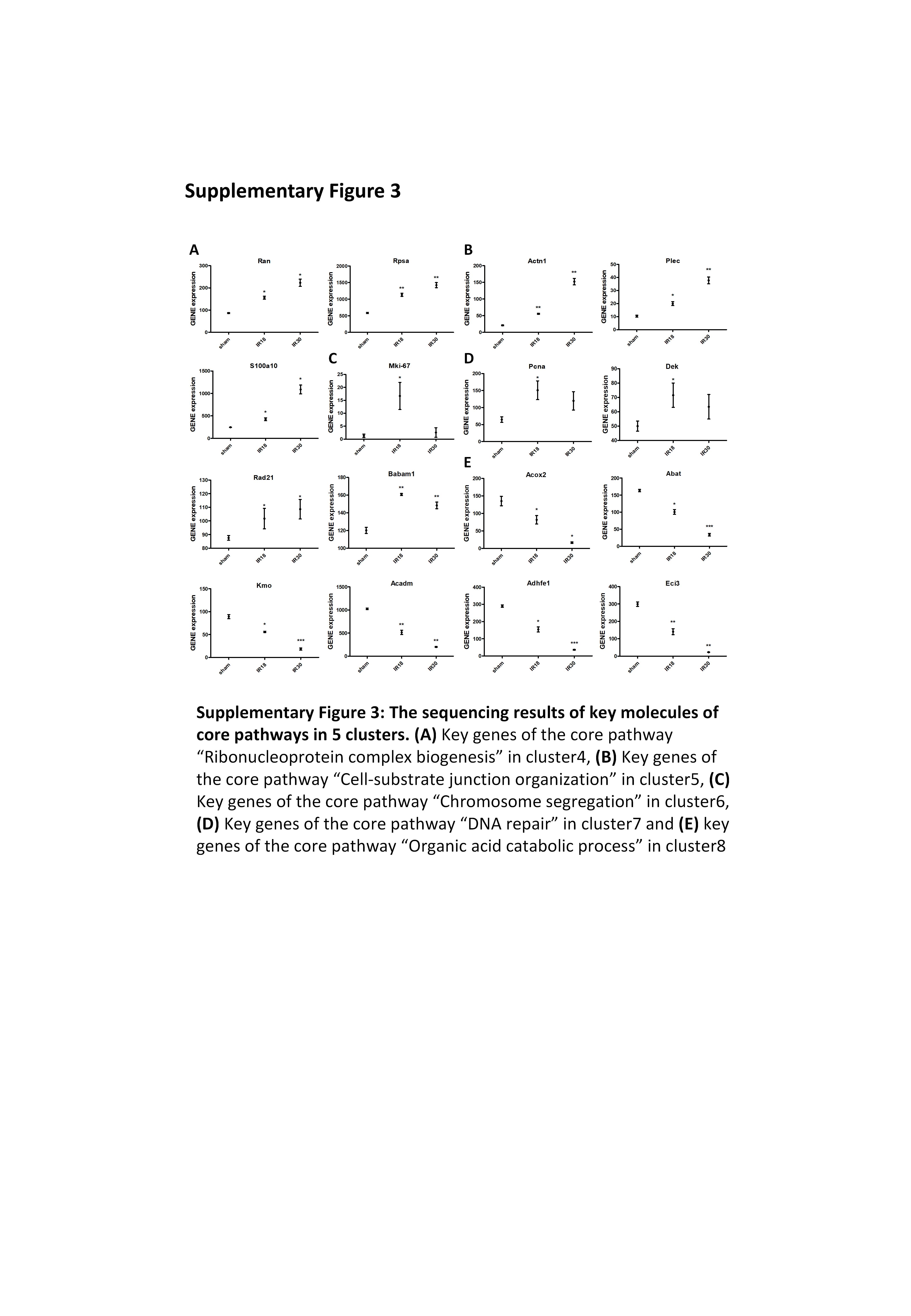

Supplement: Supplementary file 1 [file Image3.JPEG]

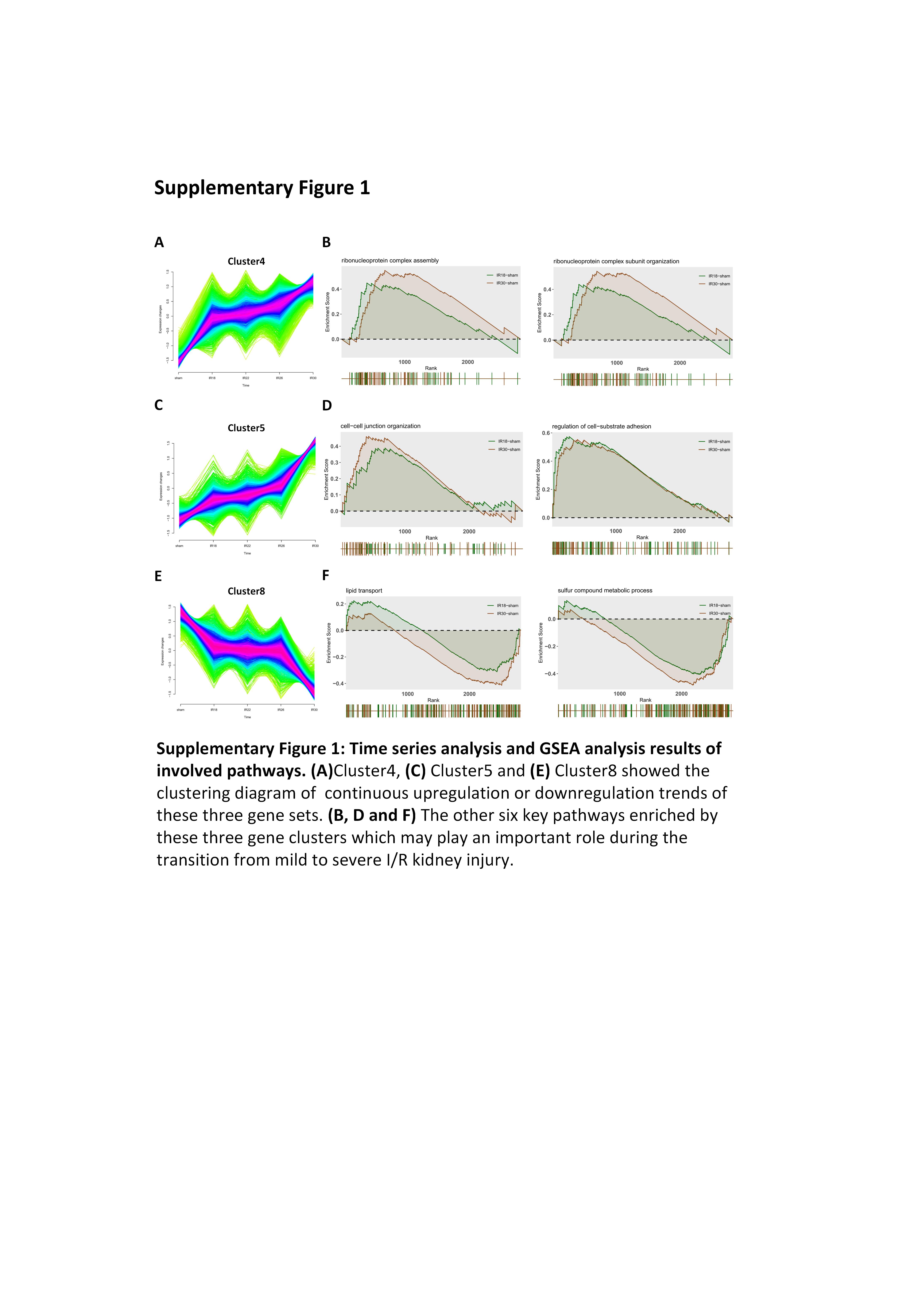

Supplement: Supplementary file 2 [file Image1.JPEG]

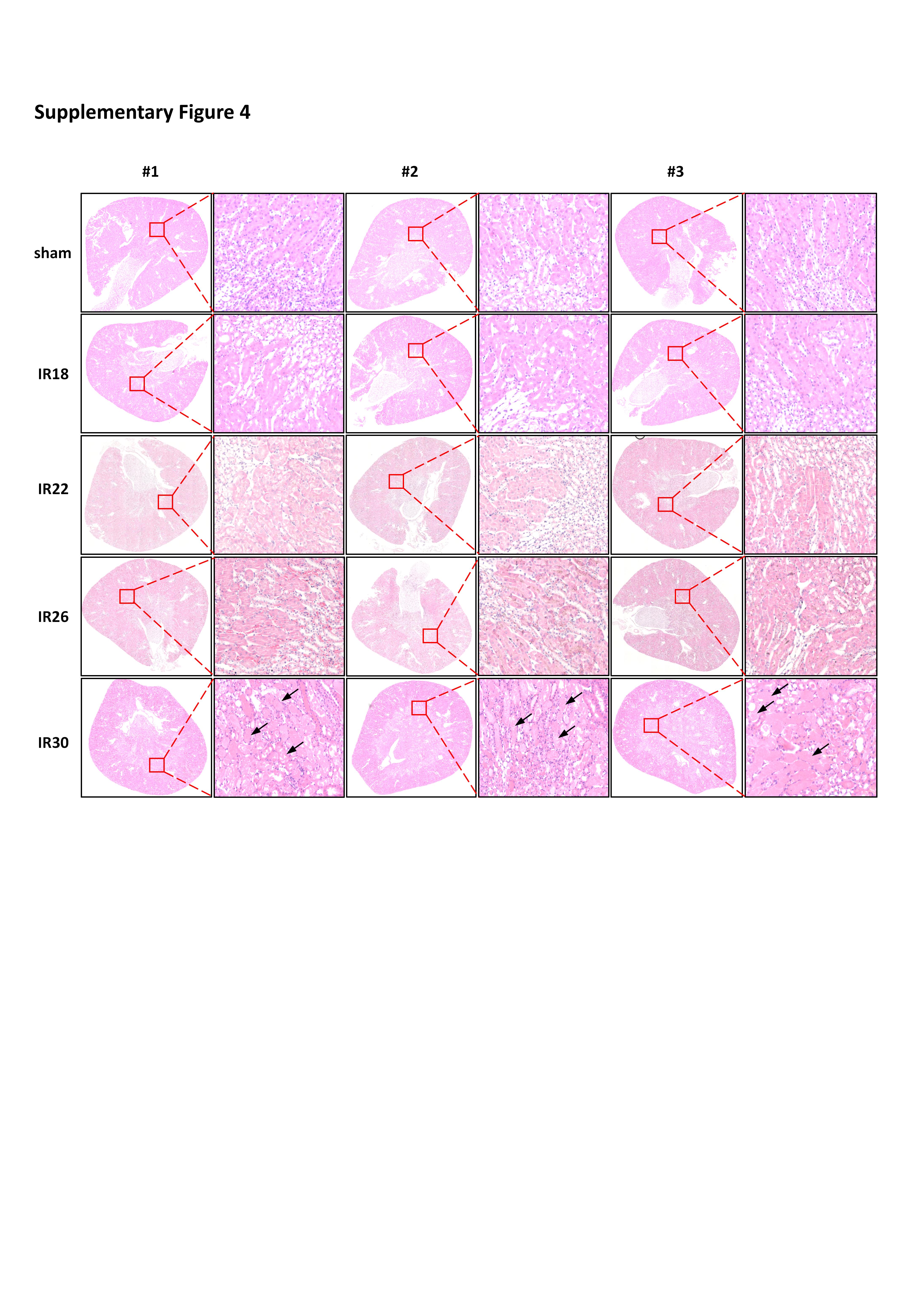

Supplement: Supplementary file 3 [file Image4.JPEG]

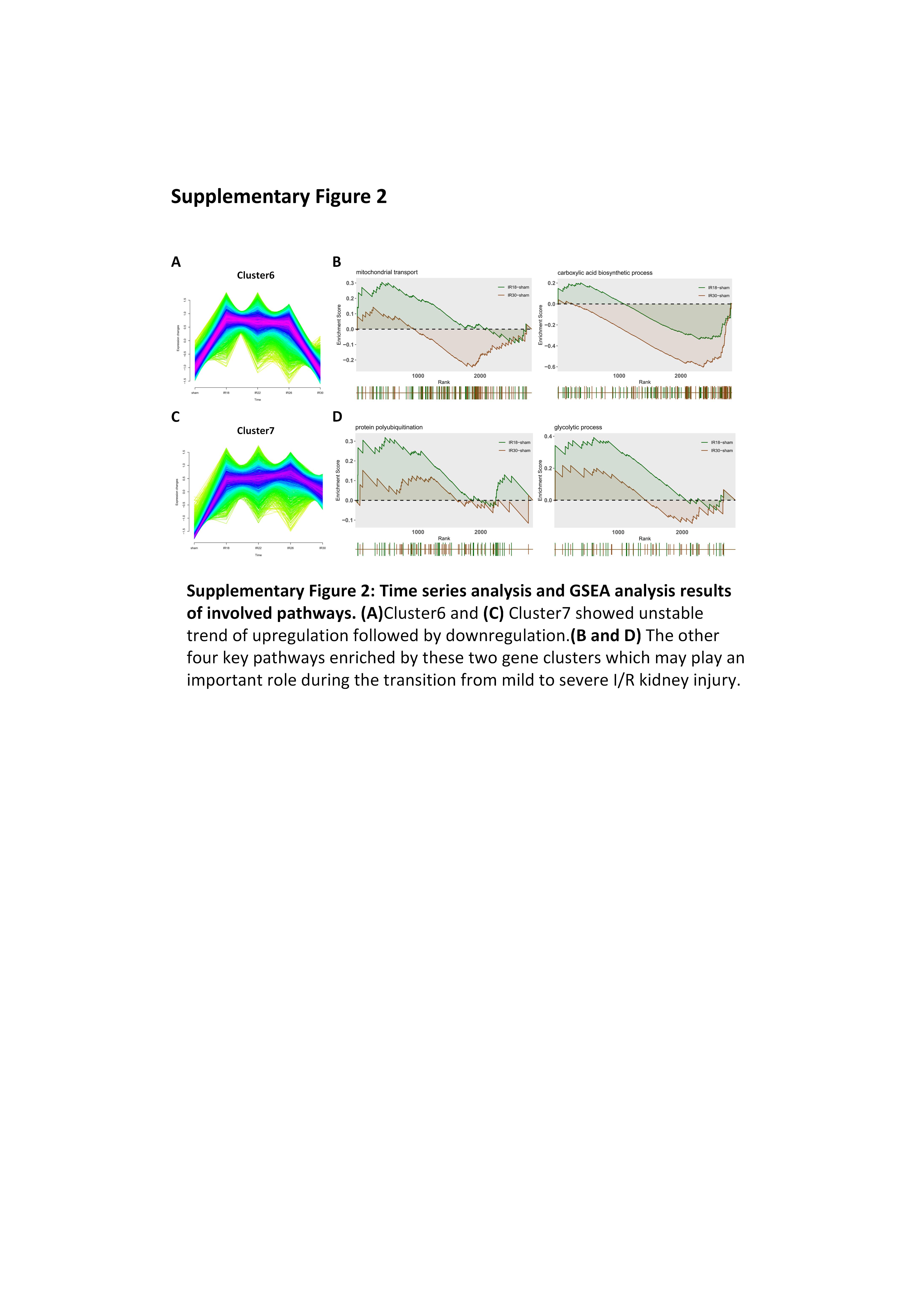

Supplement: Supplementary file 4 [file Image2.JPEG]

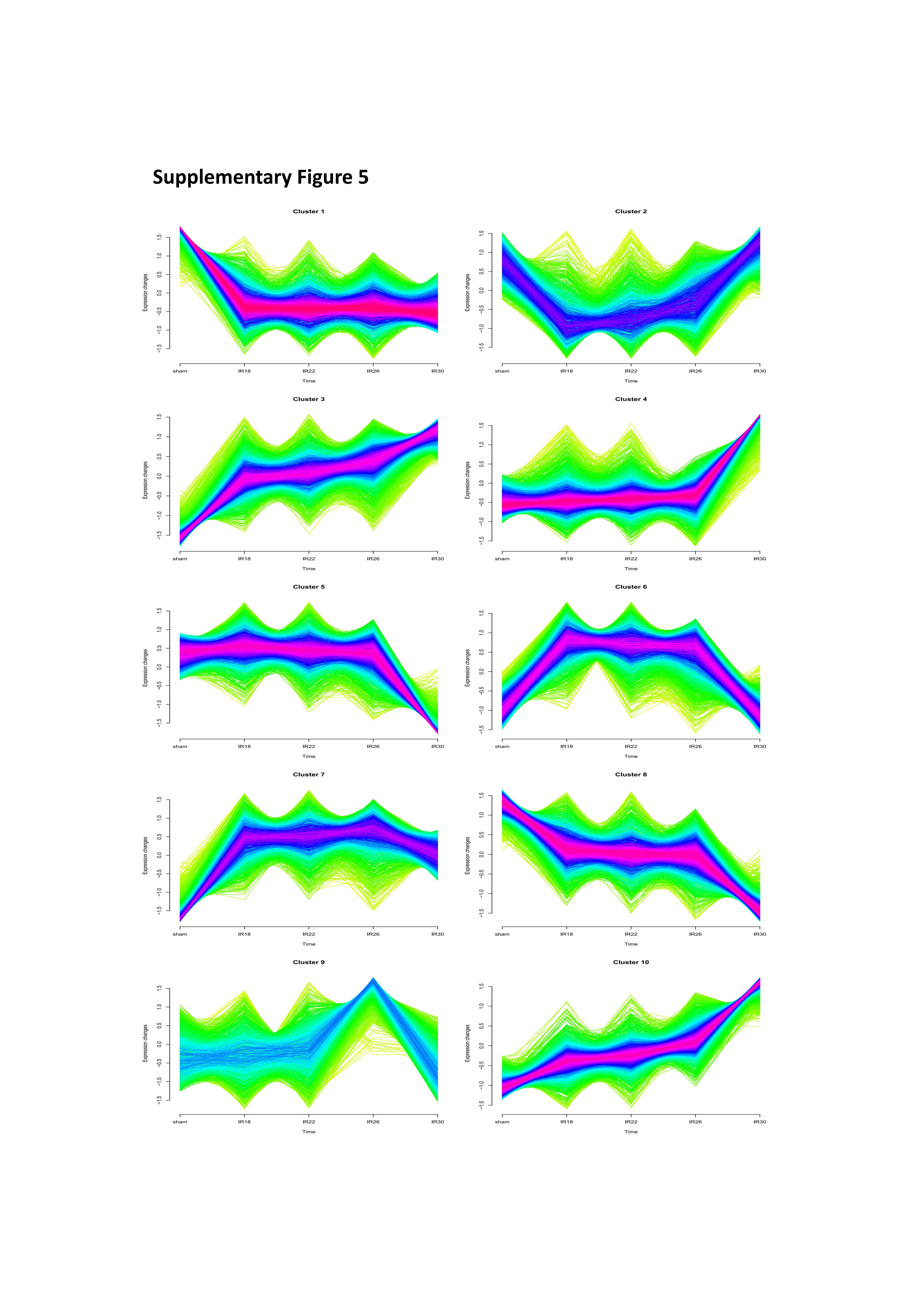

Supplement: Supplementary file 5 [file Image5.JPEG]
